# Supplementary material for: The interactome of intact mitochondria by cross-linking mass spectrometry provides evidence for coexisting respiratory supercomplexes
Source: Mol Cell Proteomics. 2017 Dec 8;17(2):216–32. doi: 10.1074/mcp.RA117.000470 (PMC5795388; doi:10.1074/mcp.RA117.000470)
Supplement: Supplemental Data [file supp_RA117.000470_133922_0_supp_23978_fzffwf.zip › spectra_annotation/mito_DR_spectra_annotation/225-1-1-1-9-1.pdf]

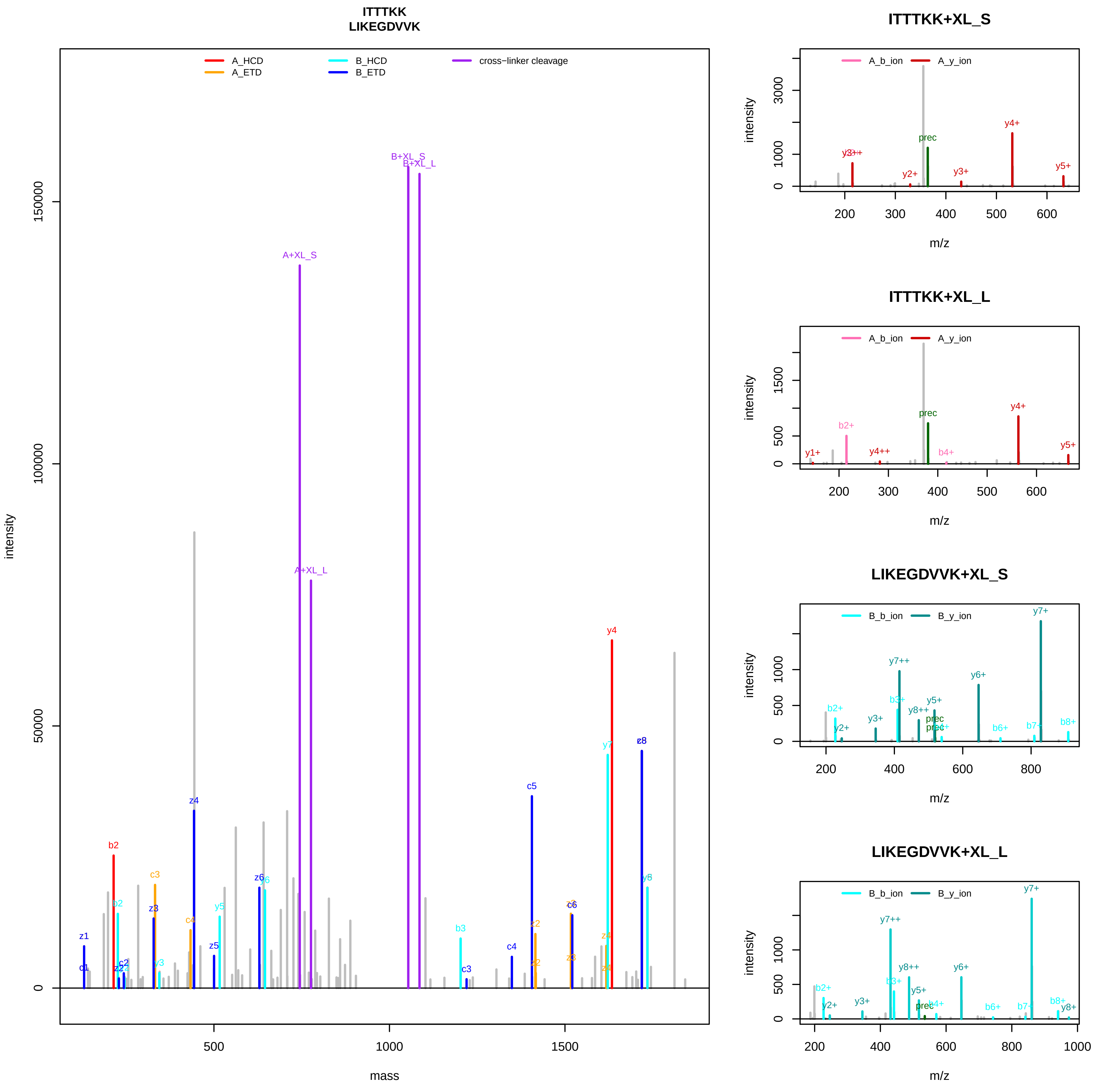
ITTTKK
LIKEGDVVK
A_HCD
A_ETD
B_HCD
B_ETD
cross−linker cleavage
B+XL_S
B+XL_L
A+XL_S
A+XL_L
y4
z8
y7
c5
z4
b2
c3
z6
y6
y8
b2
z3
c4
y5
c6
b3
z2
c4
z1
z5
c1
c2
y3
c3
intensity
mass
0
50000
100000
150000
500
1000
1500
ITTTKK+XL_S
A_b_ion
A_y_ion
prec
y4+
y3++
y2+
y3+
y5+
intensity
m/z
0
1000
3000
200
300
400
500
600
ITTTKK+XL_L
A_b_ion
A_y_ion
prec
y4+
b2+
y1+
y4++
b4+
y5+
intensity
m/z
0
500
1500
200
300
400
500
600
LIKEGDVVK+XL_S
B_b_ion
B_y_ion
y7+
y7++
y6+
b3+
y5+
b2+
y8++
prec
y3+
y2+
b6+
b7+
b8+
intensity
m/z
0
500
1000
200
400
600
800
LIKEGDVVK+XL_L
B_b_ion
B_y_ion
y7+
y7++
y8++
y6+
b3+
y5+
b2+
y3+
y2+
prec
b4+
b6+
b7+
b8+
y8+
intensity
m/z
0
500
1000
200
400
600
800
1000
